# Methyl 2-[(2-methyl­phen­oxy)meth­yl]benzoate

**DOI:** 10.1107/S1600536812005995

**Published:** 2012-02-17

**Authors:** Arun M. Isloor, U. Sankappa Rai, Prakash Shetty, Thomas Gerber, Eric Hosten, Richard Betz

**Affiliations:** a Organic Electronics Division, Department of Chemistry, National Institute of Technology - Karnataka, Surathkal, Mangalore 575 025, India; b Department of Chemistry, Manipal Institute of Technology, Manipal University, Manipal, India; c Department of Printing Engineering, Manipal Institute of Technology, Manipal University, Manipal, India; d Nelson Mandela Metropolitan University, Summerstrand Campus, Department of Chemistry, University Way, Summerstrand, PO Box 77000, Port Elizabeth, 6031, South Africa

## Abstract

In the title methyl­benzoate compound, C_16_H_16_O_3_, the mol­ecule is essentially planar (r.m.s. of all fitted non-H atoms = 0.0370 Å); the dihedral angle between the phenyl rings is 2.30 (7)°. Apart from a C—H⋯π inter­action, no marked inter­molecular contacts are obvious.

## Related literature

For the pharmaceutical background to methyl­benzoate derivatives, see: Orlek *et al.* (1991 ▶); Ankersen *et al.* (1997 ▶); Andersen *et al.* (1996 ▶).
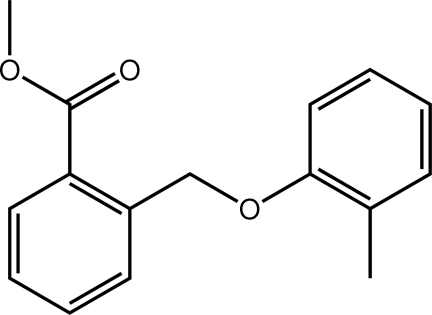


## Experimental

### 

#### Crystal data

C_16_H_16_O_3_
*M* *_r_* = 256.29
Monoclinic, 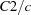

*a* = 31.6873 (13) Å
*b* = 6.5389 (2) Å
*c* = 13.8746 (6) Å
β = 111.716 (2)°
*V* = 2670.79 (18) Å^3^
*Z* = 8
Mo *K*α radiation
μ = 0.09 mm^−1^
*T* = 200 K
0.51 × 0.12 × 0.05 mm

#### Data collection

Bruker APEXII CCD diffractometer
Absorption correction: multi-scan (*SADABS*; Bruker, 2010 ▶) *T*_min_ = 0.956, *T*_max_ = 0.996
12273 measured reflections
3320 independent reflections
2295 reflections with *I* > 2σ(*I*)
*R*_int_ = 0.022

#### Refinement

*R*[*F* ^2^ > 2σ(*F* ^2^)] = 0.044
*wR*(*F* ^2^) = 0.108
*S* = 1.03
3320 reflections
174 parameters
H-atom parameters constrained
Δρ_max_ = 0.21 e Å^−3^
Δρ_min_ = −0.19 e Å^−3^

### 

Data collection: *APEX2* (Bruker, 2010 ▶); cell refinement: *SAINT* (Bruker, 2010 ▶); data reduction: *SAINT*; program(s) used to solve structure: *SHELXS97* (Sheldrick, 2008 ▶); program(s) used to refine structure: *SHELXL97* (Sheldrick, 2008 ▶); molecular graphics: *ORTEP-3* (Farrugia, 1997 ▶) and *Mercury* (Macrae *et al.*, 2008 ▶); software used to prepare material for publication: *SHELXL97* and *PLATON* (Spek, 2009 ▶).

## Supplementary Material

Crystal structure: contains datablock(s) I, global. DOI: 10.1107/S1600536812005995/tk5056sup1.cif

Supplementary material file. DOI: 10.1107/S1600536812005995/tk5056Isup2.cdx

Structure factors: contains datablock(s) I. DOI: 10.1107/S1600536812005995/tk5056Isup3.hkl

Supplementary material file. DOI: 10.1107/S1600536812005995/tk5056Isup4.cml

Additional supplementary materials:  crystallographic information; 3D view; checkCIF report

## Figures and Tables

**Table 1:** Hydrogen-bond geometry (Å, °) *Cg*1 is the centroid of the C11–C16 ring.

*D*—H⋯*A*	*D*—H	H⋯*A*	*D*⋯*A*	*D*—H⋯*A*
C25—H25⋯*Cg*1^i^	0.95	2.72	3.5461 (15)	146

Symmetry code: (i) 
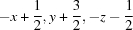
.

**Fig. 1.**
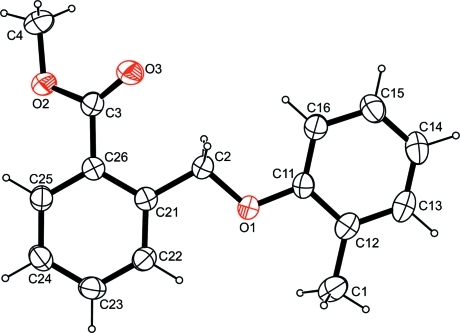
The molecular structure of the title compound, with atom labels and anisotropic displacement ellipsoids (drawn at 50% probability level).

**Fig. 2.**
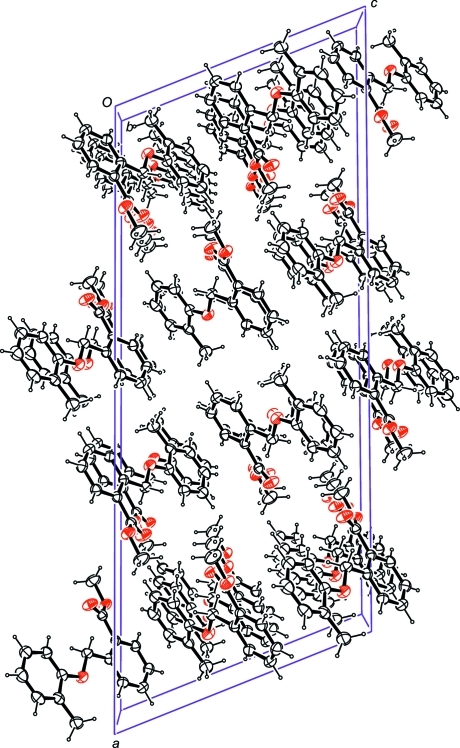
Molecular packing of the title compound, viewed along [010] (anisotropic displacement ellipsoids drawn at 50% probability level).

## supplementary crystallographic information

### Comment

Methyl 3-[(2-methylphenoxy)methyl]benzoate derivatives are extensively studied in medicinal chemistry as they are important intermediates for many pharmaceutical products. Methyl 3-[(2-methylphenoxy)methyl]benzoate derivatives are mainly used as antifungal (Orlek *et al.*, 1991) and antimicrobial (Ankersen *et al.*, 1997), diuretic, anticancer and antianaphylactic (Andersen *et al.*, 1996) agents. In view of the biological importance of the benzoate derivatives, we hereby report the crystal structure of the title compound.

The central part of the molecule, which is comprised of two connected phenyl rings, is essentially planar (r.m.s. of all fitted non-H atoms = 0.0370 Å). The least-squares planes defined by the respective C atoms of the two phenyl groups enclose an angle of 2.30 (7)° (Fig. 1).

In the crystal, a C—H···π interaction is apparent whose metrical details are summarized in Table 1. No other interatomic contacts less than the sum of van der Waals radii are observed. A view of the crystal packing for the title compound is shown in Fig. 2.

### Experimental

To a stirred solution of 2-methyphenol (1 g, 0.009 mol) in acetonitrile (20 ml) was added potassium carbonate (2.5 g, 0.018 mol) and methyl 3-(bromomethyl) benzoate (2.1 g, 0.009 mol) drop-wise. The reaction mixture was heated to reflux for 2 h. Mass analysis of the crude reaction mixture confirmed the completion of the reaction. Afterwards, the reaction mixture was concentrated and the residue was purified by column chromatography to get title compound, which was recrystallized using acetone to get single crystals. Yield: 88% (m.p. 412–414 K).

### Refinement

Carbon-bound H atoms were placed in calculated positions (C—H 0.95 Å for aromatic C atoms, C—H 0.99 Å for methylene groups) and were included in the refinement in the riding model approximation, with *U*(H) set to 1.2*U*_eq_(C). The H atoms of the methyl groups were allowed to rotate with a fixed angle around the C—C bond to best fit the experimental electron density, with C—H = 0.98 Å and *U*(H) set to 1.5*U*_eq_(C).

### Crystal data

**Table:**

C_16_H_16_O_3_	*F*(000) = 1088
*M**_r_* = 256.29	*D*_x_ = 1.275 Mg m^−^^3^
Monoclinic, *C*2/*c*	Melting point = 412–414 K
Hall symbol: -C 2yc	Mo *K*α radiation, λ = 0.71073 Å
*a* = 31.6873 (13) Å	Cell parameters from 4067 reflections
*b* = 6.5389 (2) Å	θ = 2.8–28.1°
*c* = 13.8746 (6) Å	µ = 0.09 mm^−^^1^
β = 111.716 (2)°	*T* = 200 K
*V* = 2670.79 (18) Å^3^	Platelet, colourless
*Z* = 8	0.51 × 0.12 × 0.05 mm

### Data collection

**Table:**

Bruker APEXII CCD diffractometer	3320 independent reflections
Radiation source: fine-focus sealed tube	2295 reflections with *I* > 2σ(*I*)
Graphite monochromator	*R*_int_ = 0.022
φ and ω scans	θ_max_ = 28.3°, θ_min_ = 2.8°
Absorption correction: multi-scan (*SADABS*; Bruker, 2010)	*h* = −39→42
*T*_min_ = 0.956, *T*_max_ = 0.996	*k* = −8→7
12273 measured reflections	*l* = −18→18

### Refinement

**Table:**

Refinement on *F*^2^	Primary atom site location: structure-invariant direct methods
Least-squares matrix: full	Secondary atom site location: difference Fourier map
*R*[*F*^2^ > 2σ(*F*^2^)] = 0.044	Hydrogen site location: inferred from neighbouring sites
*wR*(*F*^2^) = 0.108	H-atom parameters constrained
*S* = 1.03	*w* = 1/[σ^2^(*F*_o_^2^) + (0.0475*P*)^2^ + 1.0316*P*] where *P* = (*F*_o_^2^ + 2*F*_c_^2^)/3
3320 reflections	(Δ/σ)_max_ < 0.001
174 parameters	Δρ_max_ = 0.21 e Å^−^^3^
0 restraints	Δρ_min_ = −0.19 e Å^−^^3^

### Fractional atomic coordinates and isotropic or equivalent isotropic displacement parameters (Å^2^)

**Table:**

	*x*	*y*	*z*	*U*_iso_*/*U*_eq_	
O1	0.10397 (3)	0.81204 (14)	0.13302 (7)	0.0379 (2)	
O2	0.18815 (3)	0.10731 (15)	0.06203 (7)	0.0413 (3)	
O3	0.20550 (3)	0.42927 (15)	0.11449 (9)	0.0479 (3)	
C1	0.04466 (5)	1.1075 (2)	0.14318 (12)	0.0452 (4)	
H1A	0.0273	1.1996	0.1703	0.068*	
H1B	0.0330	0.9679	0.1401	0.068*	
H1C	0.0416	1.1517	0.0734	0.068*	
C2	0.13423 (4)	0.6661 (2)	0.11746 (10)	0.0317 (3)	
H2A	0.1505	0.5918	0.1830	0.038*	
H2B	0.1570	0.7366	0.0962	0.038*	
C3	0.17758 (4)	0.3033 (2)	0.06884 (9)	0.0305 (3)	
C4	0.23538 (5)	0.0547 (3)	0.11380 (13)	0.0519 (4)	
H4A	0.2394	−0.0923	0.1064	0.078*	
H4B	0.2451	0.0893	0.1876	0.078*	
H4C	0.2537	0.1312	0.0826	0.078*	
C11	0.12341 (4)	0.96097 (19)	0.20588 (10)	0.0311 (3)	
C12	0.09376 (5)	1.1127 (2)	0.21316 (10)	0.0331 (3)	
C13	0.11164 (5)	1.2669 (2)	0.28589 (11)	0.0410 (3)	
H13	0.0921	1.3722	0.2919	0.049*	
C14	0.15703 (5)	1.2712 (2)	0.34969 (12)	0.0443 (4)	
H14	0.1684	1.3778	0.3991	0.053*	
C15	0.18555 (5)	1.1203 (2)	0.34109 (11)	0.0411 (3)	
H15	0.2168	1.1230	0.3845	0.049*	
C16	0.16901 (5)	0.9642 (2)	0.26944 (10)	0.0359 (3)	
H16	0.1888	0.8597	0.2639	0.043*	
C21	0.10746 (4)	0.51750 (19)	0.03453 (9)	0.0282 (3)	
C22	0.06136 (4)	0.5484 (2)	−0.02189 (10)	0.0339 (3)	
H22	0.0466	0.6644	−0.0074	0.041*	
C23	0.03669 (5)	0.4130 (2)	−0.09866 (10)	0.0385 (3)	
H23	0.0054	0.4375	−0.1368	0.046*	
C24	0.05731 (5)	0.2426 (2)	−0.12015 (10)	0.0390 (3)	
H24	0.0403	0.1505	−0.1733	0.047*	
C25	0.10285 (4)	0.2062 (2)	−0.06413 (10)	0.0337 (3)	
H25	0.1170	0.0877	−0.0781	0.040*	
C26	0.12814 (4)	0.34252 (19)	0.01282 (9)	0.0277 (3)	

### Atomic displacement parameters (Å^2^)

**Table:**

	*U*^11^	*U*^22^	*U*^33^	*U*^12^	*U*^13^	*U*^23^
O1	0.0334 (5)	0.0333 (5)	0.0417 (5)	0.0048 (4)	0.0079 (4)	−0.0104 (4)
O2	0.0333 (5)	0.0335 (5)	0.0489 (6)	0.0078 (4)	0.0056 (4)	−0.0051 (4)
O3	0.0302 (5)	0.0371 (6)	0.0677 (7)	−0.0025 (4)	0.0078 (5)	−0.0114 (5)
C1	0.0420 (8)	0.0468 (9)	0.0476 (9)	0.0138 (7)	0.0173 (7)	0.0052 (7)
C2	0.0307 (7)	0.0269 (7)	0.0362 (7)	0.0029 (5)	0.0110 (5)	−0.0033 (5)
C3	0.0329 (7)	0.0299 (7)	0.0300 (6)	0.0020 (6)	0.0130 (5)	0.0000 (5)
C4	0.0368 (8)	0.0470 (9)	0.0628 (10)	0.0142 (7)	0.0077 (7)	−0.0014 (8)
C11	0.0378 (7)	0.0256 (6)	0.0311 (7)	−0.0001 (5)	0.0143 (6)	−0.0008 (5)
C12	0.0406 (7)	0.0290 (7)	0.0353 (7)	0.0037 (6)	0.0205 (6)	0.0058 (5)
C13	0.0552 (9)	0.0293 (7)	0.0485 (8)	0.0044 (7)	0.0308 (7)	−0.0012 (6)
C14	0.0562 (9)	0.0367 (8)	0.0461 (8)	−0.0092 (7)	0.0259 (7)	−0.0124 (6)
C15	0.0407 (8)	0.0407 (8)	0.0426 (8)	−0.0100 (7)	0.0164 (6)	−0.0080 (6)
C16	0.0365 (7)	0.0319 (7)	0.0401 (7)	0.0007 (6)	0.0149 (6)	−0.0037 (6)
C21	0.0310 (6)	0.0264 (6)	0.0275 (6)	−0.0008 (5)	0.0111 (5)	0.0021 (5)
C22	0.0329 (7)	0.0340 (7)	0.0348 (7)	0.0043 (6)	0.0127 (6)	−0.0004 (6)
C23	0.0277 (7)	0.0465 (9)	0.0370 (7)	0.0007 (6)	0.0070 (6)	−0.0014 (6)
C24	0.0355 (7)	0.0434 (8)	0.0345 (7)	−0.0056 (6)	0.0088 (6)	−0.0103 (6)
C25	0.0354 (7)	0.0325 (7)	0.0341 (7)	−0.0006 (6)	0.0140 (6)	−0.0050 (6)
C26	0.0293 (6)	0.0275 (6)	0.0273 (6)	−0.0014 (5)	0.0113 (5)	0.0010 (5)

### Geometric parameters (Å, º)

**Table:**

O1—C11	1.3744 (15)	C12—C13	1.3901 (19)
O1—C2	1.4251 (15)	C13—C14	1.382 (2)
O2—C3	1.3367 (16)	C13—H13	0.9500
O2—C4	1.4419 (16)	C14—C15	1.373 (2)
O3—C3	1.2034 (15)	C14—H14	0.9500
C1—C12	1.4988 (19)	C15—C16	1.3856 (19)
C1—H1A	0.9800	C15—H15	0.9500
C1—H1B	0.9800	C16—H16	0.9500
C1—H1C	0.9800	C21—C22	1.3933 (17)
C2—C21	1.5049 (17)	C21—C26	1.4054 (18)
C2—H2A	0.9900	C22—C23	1.3836 (19)
C2—H2B	0.9900	C22—H22	0.9500
C3—C26	1.4910 (17)	C23—C24	1.379 (2)
C4—H4A	0.9800	C23—H23	0.9500
C4—H4B	0.9800	C24—C25	1.3832 (19)
C4—H4C	0.9800	C24—H24	0.9500
C11—C16	1.3869 (18)	C25—C26	1.3940 (17)
C11—C12	1.3954 (18)	C25—H25	0.9500
			
C11—O1—C2	116.26 (10)	C14—C13—H13	119.1
C3—O2—C4	115.77 (11)	C12—C13—H13	119.1
C12—C1—H1A	109.5	C15—C14—C13	119.48 (13)
C12—C1—H1B	109.5	C15—C14—H14	120.3
H1A—C1—H1B	109.5	C13—C14—H14	120.3
C12—C1—H1C	109.5	C14—C15—C16	120.37 (14)
H1A—C1—H1C	109.5	C14—C15—H15	119.8
H1B—C1—H1C	109.5	C16—C15—H15	119.8
O1—C2—C21	109.13 (10)	C15—C16—C11	119.72 (13)
O1—C2—H2A	109.9	C15—C16—H16	120.1
C21—C2—H2A	109.9	C11—C16—H16	120.1
O1—C2—H2B	109.9	C22—C21—C26	118.21 (11)
C21—C2—H2B	109.9	C22—C21—C2	120.87 (12)
H2A—C2—H2B	108.3	C26—C21—C2	120.92 (11)
O3—C3—O2	122.60 (12)	C23—C22—C21	121.06 (13)
O3—C3—C26	125.65 (12)	C23—C22—H22	119.5
O2—C3—C26	111.75 (11)	C21—C22—H22	119.5
O2—C4—H4A	109.5	C24—C23—C22	120.37 (12)
O2—C4—H4B	109.5	C24—C23—H23	119.8
H4A—C4—H4B	109.5	C22—C23—H23	119.8
O2—C4—H4C	109.5	C23—C24—C25	119.80 (12)
H4A—C4—H4C	109.5	C23—C24—H24	120.1
H4B—C4—H4C	109.5	C25—C24—H24	120.1
O1—C11—C16	123.80 (12)	C24—C25—C26	120.31 (13)
O1—C11—C12	115.25 (12)	C24—C25—H25	119.8
C16—C11—C12	120.95 (12)	C26—C25—H25	119.8
C13—C12—C11	117.63 (13)	C25—C26—C21	120.22 (11)
C13—C12—C1	122.21 (13)	C25—C26—C3	118.98 (11)
C11—C12—C1	120.15 (12)	C21—C26—C3	120.77 (11)
C14—C13—C12	121.85 (13)		
			
C11—O1—C2—C21	−179.15 (10)	O1—C2—C21—C26	−172.01 (11)
C4—O2—C3—O3	0.5 (2)	C26—C21—C22—C23	−1.23 (19)
C4—O2—C3—C26	179.87 (12)	C2—C21—C22—C23	179.57 (12)
C2—O1—C11—C16	−6.01 (19)	C21—C22—C23—C24	0.7 (2)
C2—O1—C11—C12	174.45 (11)	C22—C23—C24—C25	0.5 (2)
O1—C11—C12—C13	179.82 (11)	C23—C24—C25—C26	−1.0 (2)
C16—C11—C12—C13	0.3 (2)	C24—C25—C26—C21	0.4 (2)
O1—C11—C12—C1	−0.95 (18)	C24—C25—C26—C3	−177.54 (12)
C16—C11—C12—C1	179.49 (13)	C22—C21—C26—C25	0.69 (18)
C11—C12—C13—C14	−0.4 (2)	C2—C21—C26—C25	179.89 (12)
C1—C12—C13—C14	−179.57 (13)	C22—C21—C26—C3	178.60 (11)
C12—C13—C14—C15	0.4 (2)	C2—C21—C26—C3	−2.21 (18)
C13—C14—C15—C16	−0.3 (2)	O3—C3—C26—C25	158.37 (14)
C14—C15—C16—C11	0.2 (2)	O2—C3—C26—C25	−20.99 (16)
O1—C11—C16—C15	−179.72 (12)	O3—C3—C26—C21	−19.6 (2)
C12—C11—C16—C15	−0.2 (2)	O2—C3—C26—C21	161.08 (11)
O1—C2—C21—C22	7.17 (17)		

### Hydrogen-bond geometry (Å, º)

**Table:**

*D*—H···*A*	*D*—H	H···*A*	*D*···*A*	*D*—H···*A*
C25—H25···*Cg*1^i^	0.95	2.72	3.5461 (15)	146

Symmetry code: (i) −*x*+1/2, *y*+3/2, −*z*−1/2.
